# Developing an animation-based mental health measure for children and young people

**DOI:** 10.1186/s40359-026-04036-0

**Published:** 2026-02-26

**Authors:** Victoria Christodoulides, Navya Malik, Angelika Labno, Giulia Ravaccia, Eleanor Grace, Ashton Ferguson, Omowumi Obi, Grizelda Khaling, Hotri Alapati, Melissa Cortina, Liffy McDonnell-Bond, Chloe Edridge, Julian Edbrooke-Childs

**Affiliations:** 1 Anna Freud Centre, 4-8 Rodney Street, London, N1 9JH UK; 2 Evidence Based Practice Unit, University College London, Gower Street, London, WC1E 6BT UK

**Keywords:** Mental health, Wellbeing, Image-based, Young people, Measure development

## Abstract

**Background:**

There is a need to increase the accessibility, inclusivity, and representation of mental health measures for young people, particularly for those who may struggle with traditional text-based formats due to literacy, language, or cognitive barriers. We aimed to develop an animation-based mental health measure for young people.

**Methods:**

This study involved a community-based participatory action research (CBPAR) process, including 10 four-hour in-person workshops with young co-researchers and two rounds of 90-min focus groups with different stakeholders. We also conducted think-aloud interviews with 31 young people to explore comprehension, engagement, and usability of the measure.

**Findings:**

The resulting tool, *Animi*, assesses seven symptoms of depression and anxiety through animated characters. Each scenario is depicted by an animated character (*Animi*) that indicates whether the symptom is present or absent. Users respond by selecting the animation that most closely matches their experience, using either a multiple-choice or Likert-scale format. In the think-aloud interviews, young people reported they generally understood the animations as intended and described the measure as engaging and easy to use. Feedback identified areas for improvement, like clarifying the instructions and further developing accessibility features (e.g., subtitles, customisation options). Participants also highlighted the tool’s potential relevance for younger children, neurodivergent youth, and those with additional needs.

**Conclusion:**

*Animi* represents a promising and innovative approach to mental health measurement that prioritises accessibility and co-production. Future research will revise the instrument based on feedback from the think-aloud interviews, evaluate its psychometric properties, and explore its use across clinical and educational settings. We hope this research can be used by others seeking to increase the accessibility, inclusivity, and representativeness of mental health measures for young people.

## Abstract (plain language summary)

Understanding young people’s mental health is essential; however, traditional text-based surveys can be difficult for some to use, especially if they struggle with reading, have special educational needs, or speak a different first language. To address this, researchers worked with young people to create a new animation-based mental health survey. It was developed with input from young people, mental health professionals, and researchers through co-production workshops, focus groups, and interviews.

This research reported that young people who tested this survey shared positive views, finding the animations engaging and easier to understand than traditional surveys. The new survey offers a way to make mental health research and measures more accessible, especially for those who may find traditional approaches challenging. This paper outlines the measure and the methodological processes taken to develop it.

## Introduction

Mental health difficulties for young people have been rising, with one in five young people aged 8–25 years having a mental health problem in England in 2023 [37]. It is vital to be able to understand and accurately measure young people’s mental health and respond with appropriate support. Text-based measures are the predominant way to measure and understand mental health; however, growing evidence highlights limitations of such measures in terms of accessibility, inclusivity, and representativeness.

Research on literacy and cognitive load shows that young people with dyslexia or lower reading proficiency often find reading-intensive tasks stressful and disengaging [1]. Many widely used measures are also developed in English-speaking, high-income countries and undergo only minimal cultural adaptation. A recent scoping review found that translation alone is often insufficient; although items may be linguistically correct, they are often conceptually misaligned, undermining comprehension and engagement [25]. Similarly, evidence of measurement non-invariance, where a tool does not measure constructs in the same way across cultural or language groups, has been found for widely used measures like the Strengths and Difficulties Questionnaire (SDQ) [48].

Further challenges include the sustained attention and concentration required to complete text-based measures. These demands make them less inclusive for young people with special educational needs, sensory impairments, communication challenges, or those who are neurodivergent and/or experiencing mental health difficulties [16, 34, 46]. Loss of concentration or reduced cognitive functioning may reduce response accuracy and increase biases, like acquiescence or recency bias [40, 41]. Moreover, many measures are normed primarily on majority White populations, meaning their psychometric evidence is not representative of Black and minoritised ethnic groups [48], compounding long-standing under-representation [42].

Measures that incorporate images, videos or animations may help address some of the challenges. Image-based approaches that feature visual elements in question items, response options, or both and have been shown to improve accessibility for those with lower literacy, cultural and language differences, and some cognitive impairments [15, 45]. However, two reviews of existing image-based measures did not identify any widely used image-based measures for young people’s mental health, with most existing tools utilising a smiley-face scale of smiling or frowning faces (emojis) to indicate positive or negative states [15, 45].

Along with static images, some image-based measures use videos and animations to depict the psychological constructs assessed, but these rarely use contemporary design or interactive formats [4, 5, 31]. Still, evidence suggests that image-based measures could improve representation if they are developed with and for diverse groups, yet there remains a lack of co-developed and animation-based mental health measures for young people that embed inclusivity and digital engagement from the outset [15, 45].

**Fig. 1:**
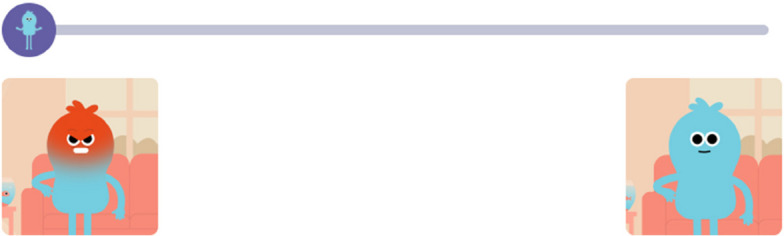
Example of a slider scale, where the user moves the scale to reflect which scenario most reflects their experiences, or which *Animi* is most like them

**Fig. 2:**
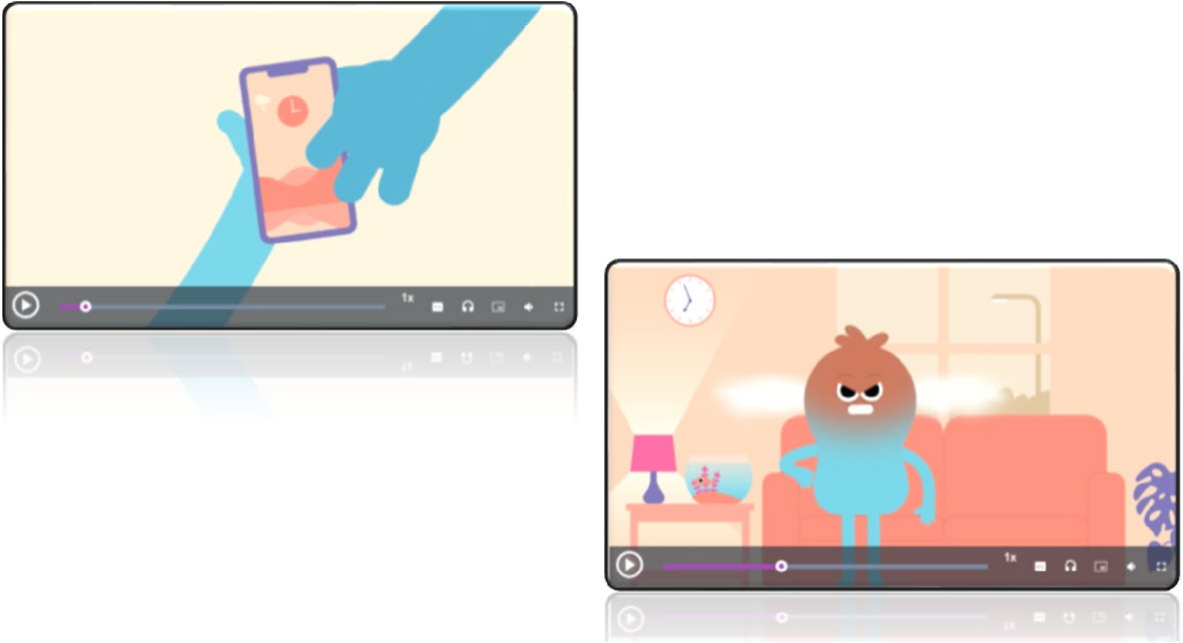
Screenshots of a video showing *Animi* having their phone taken away, which leaves them frustrated all day. The measure uses established symptoms of depression and anxiety

To address the challenges presented by current text-based and image-based measures, we co-developed a novel tool with young people. *Animi* is a mental health measure that uses short animated sequences to depict symptoms of anxiety and depression, allowing users to choose the animated character that most closely reflects their experience (see Figs. 1 and 2, which are screenshots of the measure)*.* The measure asks about seven symptoms of depression and anxiety. Each symptom is depicted through the animated character *Animi*, who experiences its presence, then its absence. Users respond either through a multiple-choice or Likert-scale format, indicating which version of *Animi* best reflects their experiences. The animated character used in the measure was named *Animi* by the young co-researchers, its meaning connected to the Latin meaning of spirit or soul. This name was chosen to reflect the tool's emotional and experiential nature. While phonetically similar to ‘anime’, *Animi* is not related to Japanese animation; instead, it symbolises internal emotional states as expressed through visual storytelling. The present research aimed to improve the measurement of young people’s mental health by creating an accessible, inclusive, and engaging tool that better reflects their experiences through a visual, interactive format.

## Methods: Phases 1 and 2

### Design

**Fig. 3:**
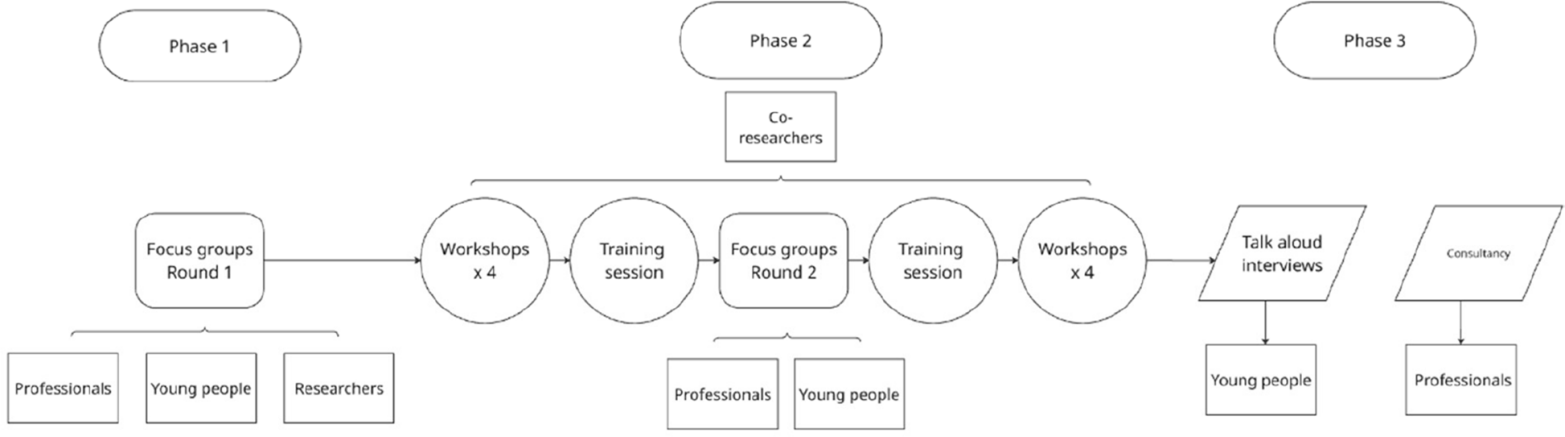
Study overview

We conducted two interlinked studies to support the development of the animation-based mental health measure over three phases (as shown in Fig. 3): Study 1 followed a community-based participatory action research (CBPAR) approach, incorporating focus groups with relevant stakeholders and co-production workshops with young co-researchers (Phases 1 and 2), and Study 2 initiated think-aloud interviews of young people using the measure (Phase 3). Each phase was underpinned by a set of research questions (RQ) described below.

### Measure overview

The tool developed through this research, *Animi,* is an animation-based measure designed with and for young people to assess experiences of mental health and wellbeing symptoms. It is unique in its co-developed animated characters, which depict both the presence and absence of key emotional or behavioural symptoms, and invite users to select the animation that most closely reflects their experience. Unlike existing text or emoji-based measures, *Animi* integrates contemporary digital animation, user preference settings, and interactive response formats developed through iterative co-production with young people. The goal was to create one of the first co-produced animation-based mental health measures prioritising accessibility, representation, and interactive engagement.

### Research questions

#### Phase 1 and 2 – Co-development phase:

- RQ 1a: What do stakeholders think are the opportunities and challenges of image-based mental health measures for young people?
- RQ 1b: What do young people from diverse groups want from an image-based measure?

#### Phase 3 – Usability and engagement phase:

- RQ 2a: What are young people’s interpretations of the animation-based measure?
- RQ 2b: What might help or hinder young people’s engagement with the measure?

The following sections outline each phase in sequence, detailing their aims, participants, and methods.

## Phases 1–2: Co-development

### Measure development framework

To structure the development of *Animi*, the research followed the initial stages of development – item development and scale development – in line with usual measure construction [35] and recommendations for developing image-based measures [45]. We did not conduct psychometric scale evaluation or validity testing in this part of the research.

### Participatory methodology

Our overarching methodology was a CBPAR design, a term often used interchangeably with participatory research or participatory action research, drawing on Lundy's [29] model of participation. These were used to align with the project’s aim to develop an accessible, inclusive, and representative measure. CBPAR is a collaborative philosophy which seeks to work *with* the communities impacted by a given challenge while facilitating empowering, knowledge-generating environments that champion the voices, decisions, and experiences of participant-researchers or co-researchers [6, 32].

As a methodological approach, CBPAR involves collaboration that seeks greater equity throughout the research cycle, including co-identification of issues, design of activities, interpretation of data, and dissemination of findings. It prioritises mutual learning and action that is directly relevant to the communities involved, especially those historically underrepresented in research [12]. The team also included a paid peer researcher, an expert by experience. The Lundy model furthers this ethically grounded approach by acknowledging the legally binding obligations of working with young people, the importance of facilitating their expression of opinions, and the significant weighting they should receive in decision-making [52].

#### Phase 1: Focus groups with stakeholders

The primary aim of Phase 1 was to gather broad stakeholder perspectives to inform the co-development process. This addressed Research Question 1a: What do stakeholders think are the opportunities and challenges of image-based mental health measures for young people? During the focus groups, participants (young people, clinicians, and researchers) were shown examples of existing text and image-based mental health measures, including commonly used tools (e.g., SDQ; [24]) and simple emoji face scales. These were used to prompt discussion about the perceived strengths, limitations, and accessibility of current tools and to elicit preferences for future designs. They then discussed their views and experiences of text-based measures; the strengths and limitations of text-based mental health measures; suggested improvements to existing measures; suggestions for what would improve young people’s engagement with the measure; and what they would like from an image-based measure.

#### Phase 2: CBPAR workshops and iterative feedback

The primary aim of Phase 2 was to address Research Question 1b: What do young people from diverse groups want from an image-based measure? We conducted workshops with young people and, to complement these and enable co‑researchers to participate in qualitative research, we returned to stakeholder focus groups midway through the project. This second round of focus groups provided iterative feedback on the developing measure, again addressing Research Question 1a. We spoke to a mix of participants, some returning from the initial focus groups and some new (eight young people, six clinicians, and two researchers).

The CBPAR workshops were delivered over five months at a central community centre in London. The group was diverse in age, gender identities, ethnicity, neuro-non-typicality, and experience of mental health difficulties (numbers not provided to avoid re-identification). Each session was facilitated by three to four facilitators and supported by accessible logistics, flyers with session and venue details, WhatsApp communications, breaks, snacks, and a meal. Information from pre‑calls about identity and accessibility needs shaped workshop delivery, ensuring the following: materials were dyslexia and neurodivergent friendly, plain language was used; slides were shared in advance; regular breaks were built in; additional facilitators offered one-to-one support where needed; activities accommodated multiple communication styles (e.g., speaking, drawing, writing, digital storyboarding).

All workshops followed an adapted version of the group-level assessment (GLA) [51], a seven-step participatory method for generating ideas, reflecting, analysing information, and encouraging action, designed to empower the championing of individuals' voices and choice within a community. These seven steps include c*limate setting, generating, appreciating, reflecting, understanding, selecting*, and *action* (discussed below). The GLA approach aligns with best practice in youth participatory research, where young people are positioned as innovators and decision-makers in co-development processes [38]. Each workshop followed iterative cycles of this process.

### Participants and recruitment

All participants were recruited through opportunistic sampling across a range of sources, including newsletters, organisations that work with young people, social media advertisements, and school contacts. Those interested completed a secure online expression of interest form. Prospective participants were contacted with the information sheet, consent form, and an invitation to schedule an online pre-call, during which they were given an overview of the study and what would be asked of them. We also verified their identity in the pre-calls to avoid including imposter participants. During the pre-calls, participants were asked to verbally report their age, location, first language, ethnicity, gender and pronouns, whether they identified as neurodivergent or had any learning needs. This ensured we could track whether our sample was representative of a diverse group of young people and highlighted any accessibility needs (e.g., providing the presentation in advance, dyslexia-friendly materials, rest breaks, or specific interpersonal support). We monitored diversity throughout recruitment and adjusted our approach when needed (e.g., reaching out to charities that supported specific groups).

Young people were eligible for inclusion if they were between 12–18 years old and lived in the United Kingdom (UK). Young people also had to have completed a mental health or wellbeing measure before to ensure the lived experience of completing a measure in real-world contexts informed our measure (e.g., completing measures at times of distress in services). Young people could only take part in one stage of the project, either focus groups, CBPAR workshops, or think-aloud interviews (see Phase 3). Clinicians and researchers were eligible if they worked in mental health for young people, lived in the UK, and had used a mental health or wellbeing measure before. All participants provided informed consent. Participant consent was sought for those aged 16 or over, while parental consent and participant assent were sought for those under 16. When this was done remotely, video calls were conducted with the child and parent/carer present to ensure both parties fully understood what was being agreed.

### Data collection and analysis

All young people were compensated with an online voucher for their time. Focus groups were recorded online or using a password-protected audio recorder and transcribed. Given the small number of focus groups, the analysis focused on summarising them, drawing on the stages of reflective thematic analysis [9].

### Ethical approval

The study was conducted in accordance with the Declaration of Helsinki and received ethical approval from the University College London (UCL) Research Ethics Committee (14037/013).

## Results: Phases 1 and 2

### Focus groups: round 1—challenges with text-based measures

We conducted three 90‑minute online focus groups with six young people, four clinicians, and eight researchers to explore initial views on image-based measures and gather ideas to take forward into the CBPAR workshops. All three groups discussed the challenges with text-based measures, highlighting that they are often too long, repetitive, wordy, and dull. Practitioners particularly felt that text-based measures were less accessible, as they assume a high level of literacy and are hard to understand or interpret. Young people discussed that questionnaires are often vague, time-consuming, and boring. They questioned whether or not their feelings were meaningfully captured and understood.

Despite these weaknesses, the groups identified several strengths that could be applied to an image-based measure. All groups valued accessibility and agreed that the measure should be easy to access and complete. They also wanted an easy way to see and share a summary of the responses. Both young people and practitioners discussed the importance of depth and the opportunity to provide more detailed answers. Both practitioners and researchers discussed how measures can help develop a therapeutic relationship. Practitioners also highlighted the importance of a mental health measure being disorder- or symptom-specific and showing progress or change over time.

### Desirable features of image-based measures

The groups’ suggestions for improving measures and young people’s engagement included keeping measures short and straightforward, providing clear instructions, ensuring access to phones/tablets to complete them, and ensuring measures are meaningful to young people. The main considerations flagged were the relevance of images (e.g., identifying with images, relevance to younger vs. older children, relevance over time).

When discussing what they would like from an image-based measure, participants said that young people should be able to choose between images and text. Young people felt that an image-only measure might be too vague and that older children may prefer text to images. The groups also wanted young people to be able to customise their response options, like adding an audio option or choosing their own images and colours. To increase engagement, participants said the measure should include an element of gamification or interaction, like creating a main character that represents the responding individual. Everyone also agreed that it should be easy to see their responses over time and suggested using graphs or trackers to do so. Practitioners said this was essential for comparing before- and after-treatment outcomes.

### Next steps

The insights on challenges with text-based measures and desirable features of image-based measures informed the next stage of the research. During the CBPAR workshops, discussed below, we used these insights to help contextualise the project with young co-researchers. The insights helped provide the young co-researchers with a snapshot of broader perspectives beyond their own.

### CBPAR workshops (Phase 2)

We conducted 10 four-hour in-person CBPAR workshops with 10 young co-researchers (see GRIPP2 checklist).

### Activities

In line with the first step of the GLA approach, *climate setting*, workshop 1 established a ‘relationship partnership’, creating a group agreement about communication, how to navigate tensions or disagreements, hierarchical challenges, session expectations, and aims (e.g., [12, 29]). The agreement was reviewed at the beginning of each session and adjusted if necessary.

In addition to the ongoing *climate setting* in the workshops, co‑researchers conducted asset-mapping exercises to identify individual and group skills or experiences that could be relevant for the project [49]. This was carried out by using an A4 piece of paper with an outline of a body, which asked each co-researcher to draw or write what these skills, experiences, or values were [22]. Consistent with the second step of the GLA approach– *generating*, the young co-researchers reviewed findings from the initial focus group and explored examples of existing image-based measures.

**Fig. 4:**
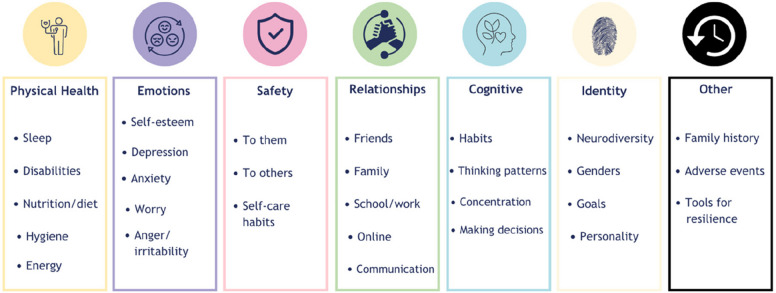
Domains identified by young co-researchers

Workshops 2 to 4 focused on identifying and refining domains for the measure using World Café dialogues and collaborative mapping of emerging domains against diagnostic criteria for depression and anxiety [3]. The World Café approach is a structured conversational process designed to foster collaborative dialogue and share collective knowledge through small, rotating group discussions around pre-determined questions [10]. In line with the third, fourth, fifth and sixth steps of the GLA approach, *appreciating, reflecting, understanding* and *selecting,* co‑researchers decided which domains to develop further, dropping those too complex for animation. These domains are shown in Fig. 4 and were compared with the focus group findings on desirable features of image-based measures, which were subsequently mapped to the diagnostic criteria for depression and anxiety [3].

### Key outcomes—domains identified by the young co-researchers

Consistent with the GLA approach, the steps of *understanding* and *selecting*, and mapping the initial domains (Fig. 4) onto the diagnostic criteria for depression and anxiety, resulted in eight additional domains to be further developed: sleep, energy, decision-making, concentration, irritability, worry, somatisation, and eating behaviours. Later in the workshops, somatisation and eating behaviours were discontinued due to the complexity of turning them into animations within the project's timeframes. In line with the seventh step of the GLA approach, *action*, alongside measurable domains, other notable elements were championed by the young co-researchers for the measure, like: personalisation of the tool, agency over their data, flexibility of use, and incorporation of coping mechanisms and information. All elements, bar coping mechanisms, were incorporated into the measure design in some way at this stage, with further elements to be developed in subsequent phases.

Before the next session, and in keeping with the GLA step-*climate setting,* young co-researchers requested that we do a team-building exercise (i.e. bowling) to develop relationships with one another. Workshops 3 and 4 followed a similar structure to workshop 2, following the steps of *appreciating, reflecting, understanding* and *selecting,* to further refine the domains. Accordant to *generating*, sessions would involve a cumulative approach to developing ideas. Young co-researchers were each provided a computer and, in pairs, invited to choose one of the domains to design or refine using digital storyboarding on an online interactive whiteboard. This platform featured various GIFs (animated images), symbols, and objects for young co-researchers to use, providing flexibility. Digital storyboarding is gaining increased attention within CBPAR projects, facilitating accessible ways for individuals to create information in engaging ways that relay their ideas and perspectives [27]. The storyboarding also enabled the group to work collaboratively. In a process of *appreciating, reflecting, understanding, selecting*, and *actioning*, storyboards were then collectively shared, and decisions were made about which elements would be taken into production.

### Measure development – moving from an image-based measure to an animated measure

During the storyboarding sessions, in line with the GLA approach's steps of *appreciating* and *reflecting*, it was observed that the young co-researchers used GIFs to convey their concepts. The purpose of which was further explored as a group, consistent with the step of *understanding*. Young co-researchers found moving or animated elements easier to use to represent their ideas. Following the steps of *selection* and *action*, this led to a decision to move from still images to animations, shifting the project's initial plans, which were directed by the young co-researchers. Young co-researchers led the design of the animated main character ‘*Animi’*, featuring in all animations of the constructs assessed in the measure. Young co-researchers collaboratively decided on the gender-neutral features and name of the animated character, aiming to ensure it was as inclusive and representative of all potential young users of the measure as possible. In addition, young co-researchers not only guided the development of the animations’ narratives through digital storyboarding but also the design of the animations’ settings. The lead technical developer of the online platform on which the measure was being developed also supported young co-researchers in sessions to outline the measure's structure within the platform. Outside of the sessions, we worked with an animator to produce the animations.

In line with the CBPAR approach, to support their research involvement in workshops 5 and 6, young co-researchers were provided with training in research methods, including how to design and deliver focus groups and conduct thematic analysis [7]. The young co-researchers then co-developed and co-delivered the second round of focus groups (discussed below). During this period, the technical developer produced mock-ups of the measure, and the animator produced initial animations.

### Changes to the measure

Informed by feedback from the focus groups, and following the iterative process of *generating, appreciating, reflecting, understanding,* and *selecting*, in workshops 7 to 9, young co-researchers worked in pairs on an online interactive whiteboard, reviewing animations and the measure as it was being developed on the online platform. The young co-researchers provided feedback on the design and comprehension of the animations, and changes were collaboratively agreed upon and discussed with the animators to implement. The young co-researchers worked with the lead platform developer at all stages of development, from reviewing mock-ups to providing feedback on prototype components of the measure as it was being developed, thereby shaping the measure's design.

The final workshop presented the finalised prototype and celebrated the team's achievements. Reflexive analysis was utilised throughout the CBPAR process to enhance its participatory and critical aspects (e.g., [12]). This principle strengthened the ethical and collaborative foundations of CBPAR by ensuring that the researchers and young co-researchers engaged in mutual reflection and co-construction of knowledge. The workshops produced the final six measure domains (i.e., sleep, energy, decision-making, concentration, irritability, and worry); generated animation narratives developed using digital storyboarding; and designed the animations’ settings, as well as those of the main animated character, ‘*Animi’*.

### Focus groups round 2

The second round of focus groups was held to review the mock-ups of the digital measure designed by the young co-researchers and the initial animations. The topic guide was co-developed by three researchers, the peer researcher, and the young co-researchers. Young co-researchers facilitated the focus groups, took anonymised notes, or observed the sessions. Focus group participants included both first-round and new participants. They viewed a mix of animated videos and still images from the measure and provided feedback on the animations' clarity and areas for improvement. In general, young people liked the concept of the animation-based measure, the specificity of items, and the response scale. Practitioners and researchers also viewed the measure positively; however, they acknowledged challenges in capturing nuances in behaviour through animations and in finding images relevant to all children. Some young people found the animation's still images clear and easy to understand. Yet others found giving feedback on still images confusing, as “seeing them one by one” rather than the full animation made it “hard to understand the process” of developing the animation-based measure.

### Emotional expression and clarity

Regarding clarity, practitioners and researchers found the messages in the animations relatively clear. In contrast, young people said the “facial expressions [of the animation] are not clear enough” and might be “throwing people off”. Young people suggested that the different facial expressions could be more distinct from one another. For example, for the irritability question, young people highlighted that “there’s not much of a distinction between one character getting slowly angry at a situation and a character getting quickly angry at a situation” and that it would be clearer “to have more of a neutral look for the slow to anger one”. Emojis were also suggested to clarify *Animi’s* emotions. Professionals and researchers offered several suggestions to improve animation clarity. One suggestion subsequently incorporated into the measure was adding more body cues (e.g., furrowing and softening *Animi’s* eyebrows) to better illustrate “irritability.” The research group also suggested adding an image to represent disrupted sleep, which was added to the ‘Sleep animation’ response options as “broken sleep”. Another suggestion was to include subtitles in different languages.

### Accessibility

Regarding the accessibility and suitability of the measure, one young person felt it could cater to people with “different personalities” because it encourages people to acknowledge that “issues with mental health don’t only involve the emotion of sadness”. Conversely, the researchers were concerned about how accessible the animations would be to non-verbal children who might find them more difficult to understand. A few participants in the professional and researcher groups suggested stick figures may be more relevant and recognisable for use with neurodivergent populations.

### Relevance to young people

Professionals and researchers commented on the relevance of the animations and their settings to the populations with whom they work. For example, one professional felt the measure would be a good tool to support children at risk of suspension and noted that its “modern” feel would be more relatable to children. However, a couple of young people expressed that the animation's still images did not “really capture each individual’s feelings and how they cope or respond to different situations”. Indeed, one young person explained that “people may react in different levels of severity, which isn’t completely recognised”. Young people, therefore, suggested changing the scenario to one that other young people could relate to better.

### Customisation

Practitioners and researchers expressed a desire for further customisation to allow for cultural and personal relevance. For example, the groups had mixed opinions on *Animi*, with some liking the characters and others preferring different avatar options for young people to choose from or create. Moreover, customising their background environments (e.g., school, home, park) or including different activities (e.g., star jumps instead of riding a bike to represent energy levels) were suggested.

### Summary

Overall, young people, practitioners, and researchers liked the animations and still images and provided feedback to refine their clarity, specificity, and relevance. For example, additional features (e.g., furrowed brows) were added to animations to improve the clarity of emotional expressions. Moreover, further options were added to some items (e.g., broken sleep). Suggestions for increasing comprehension, accessibility, and relevance incorporated the inclusion of emojis, subtitles, and user-customised backgrounds and activities.

## Methods: Phase 3

### Phase 3 – Think-aloud interviews

#### Design and purpose

Phase 3 represented the second study in this project, following on from the development work undertaken in Phases 1 and 2 (see Fig. 3). This phase involved think-aloud interviews with a new group of young people who had not participated in earlier phases. The aim was to evaluate *Animi*’s usability, clarity, and inclusivity, addressing Research Questions 2a and 2b.

#### Participants and recruitment

Recruitment and eligibility procedures mirrored those used in Phases 1–2. Young people were eligible if they were aged 12–18 years, lived in the United Kingdom, and had prior experience completing a mental health or well-being measure. Participants were recruited through schools, youth organisations (including Mind), and social-media advertisements. We also worked with one school that offered both mainstream and specialist provision, helping to identify and reach participants across a range of ages, neurotypes, gender identities, and cultural backgrounds. Interested individuals completed an online expression of interest form and participated in a pre-call during which researchers explained the study, answered questions, and ensured accessibility needs could be met. Because several sign-ups came from individuals outside the UK or outside the eligible age range, all prospective participants were required to register through a secure sign-up form and complete an identity-verification pre-call before scheduling their interview. Each participant received a £10 voucher for their contribution. Following recruitment and consent procedures, participants took part in online think-aloud interviews to explore how young people engaged with, interpreted, and experienced the *Animi* prototype measure.

#### Procedure and data collection

Think-aloud interviews were conducted online with 31 young people, facilitated by two researchers and lasted 30–45 min. The interviews aimed to assess comprehension, inclusivity, and engagement with the *Animi* measure. The think-aloud method [20] gathers participant perspectives and thought processes while performing a task and typically reveals more usability issues than retrospective methods [36]. However, as this approach relies on working memory, it can influence what key factors participants recall during the task [13, 20]. To address this, a retrospective questioning session followed the think-aloud process to triangulate and expand on the collected data.

A semi-structured interview schedule informed by previous research involved three stages: preparation, delivery, and semi-structured retrospective questions [43]. As the interviews were conducted online, participants were asked to share their screen so the facilitators could see their actions as they completed the measure.

*Animi* consisted of seven questions, divided into two sets (i.e., Q1-3 and Q4-7), assigned across participants to allow adequate time to review. To assess comprehension, participants first completed their assigned questions without text, subtitles, or audio. Then they selected user-preference options (e.g., background colour and pattern, dyslexic font, subtitles, audio description, text box, hover box) and reviewed the same questions again. Finally, retrospective questions explored participants’ views on the content, usability, design and potential contexts of use (e.g., school, community, clinical settings).

#### Data analysis

Collaborative thematic analysis was applied to the interviews [44]. Two researchers independently coded four sample interviews,these codes were used to collaboratively develop a codebook, which the four researchers then applied to all interviews. Regular meetings supported iterative code refinement and consensus building, promoting reflexivity and analytic rigour throughout the process.

## Results: Phase 3

We examined two research questions: 1) What are young people’s interpretations of the animation-based measure? and 2) What might help or hinder young people’s engagement with the measure? Overall, young people reported liking the measure and found it fun and engaging. Generally, they understood the main questions being asked and interpreted what was being conveyed in line with expectations. The relevance of the animations to young people in general helped with engagement. There were suggestions for increasing the clarity of some of the main questions. The instructions for answering the question, which appeared as an animated video before each question, were described as unclear when watched without audio and/or subtitles. Young people described a range of populations for which the measure could be used, including younger age groups, those with additional needs (including neurodivergent young people) and those with less familiarity with English. They also noted that it could be used across a range of settings. The main group whose access would be hindered was those in digital poverty; however, young people offered recommendations for paper-based options.

### RQ 2a. What are young people’s interpretations of the animation-based measure?

Overall, participants liked the animation design and the measure's aesthetics. They appreciated the use of colours, noting that they were “not overwhelming” (Interview 2), “engaging” (Interview 24), and “calming” (Interview 7). Most participants had positive emotional reactions to the measure, including laughter and described it as “fun” or “interesting” to look at. This included laughing in response to *Animi* or the video’s sound effects. Many participants referred to *Animi* and the characters as “cute” and “fun”. They liked that *Animi* was an animated character rather than a real person, and one participant reported that this made it easier to relate to and understand the cartoon. A few participants particularly liked *Animi's* consistency as a main character throughout the measure’s questions. Participants noted that the animated characters made the videos more enjoyable and engaging than if real people had appeared. One participant observed that this “can make people feel safer and more willing to share more.” (Interview 22).

There were mixed opinions on speed and length, but the videos were overall considered short and well-paced. Participants generally liked the response options; however, they suggested a clearer middle option for the slider response scale, as participants could not select an exact middle point. A few participants also wanted to see an option for “unsure” or “other”, and the ability to expand on that. There were a few instances in which young people suggested alternative ways to respond, like multiple-choice or adding anchors to the scale (e.g., from very easy to very difficult).

### Interpretation of animations

Participants reported that animations helped them focus on the questions more effectively than text-based questionnaires:

“I think with all the words, I overthink it. Sometimes, I can’t quite get exactly what it’s asking me about. But I feel like with the images showing the facials and everything like that, you can kind of get more what it’s trying to ask.” (Interview 18).

Young people’s interpretations of the animations and the questions were generally consistent with the intended questions. For example:

“Like it's asking you like, is it hard for you to focus or is it easy for you to focus?...Because this man, the one on the, the left side, he was distracted by everything, and his card tower fell down. And the image on the left, uh, the right was, he was not like getting distracted by everything. And his card tower didn't fall down.” (Interview 11).

However, there were a few instances in which young people interpreted the animations differently from how they were intended. One young person, for example, thought the animation about concentration was reflecting general life satisfaction: “Some people have better lives than others, I would say, because the one on the left obviously isn't having fun doing what he's doing, but the one on the right seems pretty content.” (Interview 22). Another young person interpreted the items representing worries as reflecting concerns about material possessions rather than worries.

### Clarity of animations

The audio/subtitles were generally described as making the animations clearer, even though the main questions could be understood without them. This was described as particularly relevant for younger age groups, for example:

“The animation made sense itself, but again, some young people, slightly younger, same age, I don’t know, still might’ve found it a little harder to deduct what was going on... But, with the subtitles, that’s really clear. That’s really good.” (Interview 18).

Additionally, making *Animi’s* expressions more exaggerated was suggested as helping to make the animations clearer without using audio/subtitles:

“I think I think it's a bit unclear on what like it's actually trying to show…But maybe if you made the guy on the left a bit more upset or like really struggling. Rather than him just like contemplating…And the guy on the right like confidently making the decision and showing that he made the decision really easily.” (Interview 13).

However, other young people viewed the expressions to be clear, for example: “I really struggle with identifying facial expressions with the autism. But even for me, I thought that was really clear.” (Young person 2). Several participants suggested using more non-verbal communication and gestures (e.g., thumbs up) to increase accessibility for respondents who do not speak English or are less comfortable with it.

The relevance of the animations to young people in general and to individuals personally shaped how they made sense of each scenario. In some instances, this led to meanings that diverged from the intended emotional construct. For example, the animation depicting a phone being taken away, intended to represent anger, was interpreted by one participant as signalling worry, “thinking too much about everything,…which, to be honest, I experience that a lot sometimes” (Interview 16). In contrast, other animations were read more in line with the developers’ intentions. When viewing the scenario in which *Animi’s* headphones break, one young person explained:

“when the person's headphones break and they're mad all day, which is exactly what I'd be. And then the second one, they don't stay cross all day. Which good on them, could not be me.” (Interview 9).

One young person suggested the sleep animation could be more relevant by including a scenario where a young person goes to bed but then spends a lot of time on their phone rather than sleeping:

“sometimes I come upstairs and like, “Okay, I’m going to get ready for bed.” And then, like an hour later, I’m like, “Well, I’m still not ready for bed,” I’ve just been sitting on my phone.” (Interview 30).

Several young people commented on the consistency of the narrative within and between videos. For example, in the animation about energy levels, *Animi* cycles to school and then takes a bus, which was described by some as confusing. Some young people commented on the relationship between sleep and subsequent videos, such that it impacts energy levels throughout the day. One young person remarked that this was similar to text-based questionnaires they had completed, which asked about similar concepts but from different angles:

“even in just normal questionnaires that I’ve done at school...There are multiple questions that you’ll have the same, “Rate this from one to four,” and the same boxes. And they’re different questions; they might be for a different purpose or use.” (Interview 30).

### Customisation

Several young people made suggestions for customising the animation, which may help to increase the personal relevance of the measure. Examples included choosing the voice for the audio option, the ability to select from a single scale response to a multiple-choice response, choosing other languages than English, having a dark-mode option, reducing the noise in the videos for the with-sound option, and choosing their own *Animi*:

“maybe to add, was, maybe if people wanted to choose their own *Animi*, was it. Yeah, if they wanted to choose their own like little person to say it.” (Interview 5).

### Instructions video

Young people expressed a need for further clarity in the instructions video, and some found the repetition of the instructions for each question irritating. For example, they found it hard to understand without audio/subtitles, which caused confusion and frustration: “The video with the instructions was a bit confusing, but the actual video where it's like this person’s this, this person’s that, that made perfect sense.” (Young person 9). Several young people suggested expanding the instructional video to show *Animi* completing the entire measure, rather than just showing users how to complete the response options or giving users the option to turn off this explanatory video.

### RQ 2b: What might help or hinder young people’s engagement with the measure?

Young people identified several settings where the measure may be suitable, as well as considerations for whom its use would be most appropriate. Settings included schools, with “school counsellors” (Interview 19), “during a PSHE lesson” (Interview 23) or a “life skills class” (Interview 31), or as a replacement for “SEND [special educational needs and disabilities] surveys and stuff at school… as the monster was showing you what to do instead of the teachers having to explain it”. (Interview 27). They also felt that the measure could be helpful within mental health settings, for example, as “part of assessments” (Interview 1), as “if you go to CAMHS [child and adolescent mental health services] … sometimes you get given the sheets to do every week… I think, having something that's a bit more engaging to do each week would just make that easier”. (Interview 2).

### Suitability for different groups

Participants had varying views on whom the measure would be suitable for. While some participants felt that as the measure was “easy for everyone to use” (Interview 22) and people across a range of ages could use it, a large majority agreed that the measure would be most suitable “for younger kids” (Interview 2) because of its “images and cartoons” (Interview 30) and “simplistic designs”. (Interview 13). Whereas some felt that older young people might “not take the character seriously” (Interview 18), as they might perceive it as being “a bit babyish”. (Interview 25).

### Accessibility

Accessibility was also a major consideration for young people when asked who the measure would be suitable for. There were conflicting views on its utility for those with hearing impairments and those who are unable to read or do not use English as their first language. Some young people noted that “you have the subtitles for people who may not be able to hear” (Interview 5), and “in the video, it doesn’t have the characters say something.” (Interview 14). Still, others pointed out that the measure might be “hard to understand” (Interview 7), “even with all the images”. (Interview 30). Similarly, a few participants mentioned that those with visual impairments would have difficulty using the measure and offered suggestions like a zoom-in function or “an auditory setting where you can click on things, and it reads it out for you”. (Interview 7). A few stressed the importance of finding preference options easily:

“So, I really do think this should be the first thing that pops up because let’s say someone deaf or had a hearing difficulty did come into this questionnaire, couldn’t understand anything, it would just be a bad response to everything”. (Interview 21).

Young people also considered those without access to digital technology, with one suggesting the measure “could be in like a written like printed out form”. (Interview 17). The measure was also seen as being inclusive of people who are neurodivergent or have disabilities, e.g., “there could also be children with autism, or ADHD [attention deficit and hyperactivity disorder], or something, and it definitely catches their eye”. (Interview 25). Likewise, quite a few young people appreciated the preference options that allowed them to make font and colour adjustments, as “the dyslexic [font] is, I find it's easier to read than some other fonts”. (Interview 13). Lastly, participants described the gender inclusivity of the measure as positive, e.g., “I really like how it's 'they'. I think that that is so much more inclusive”. (Interview 2).

### Summary

Overall, the think‑aloud interviews showed that most young people could interpret the animations as intended and found the tool engaging and easy to use. Participants highlighted specific areas for improvement, including clearer instructions, improved accessibility options (e.g., subtitles, dyslexia‑friendly fonts), and the need for culturally and personally relevant scenarios. They also offered feedback on the tool's technical aspects, including preferences for layout, navigation, and video pacing. This feedback informed several refinements to *Animi* and will guide further development, particularly in terms of inclusivity and usability.

## Discussion

### Rethinking youth mental-health measurement

Traditional mental health questionnaires for young people have relied mainly on lengthy, text-based instruments, a format that, while established, carries well-documented limitations (e.g., literacy demands, cultural/language bias, cognitive load) ([23], Christodoulides et al., in press; [45]). Our work responds to calls for more inclusive, engaging, and accessible assessment formats by co-developing an animation-based measure, *Animi*, designed with and for young people. By centring youth voices through participatory design (Phases 1–2) and evaluating usability via think-aloud interviews (Phase 3), this study provides initial indications that animation may support accessible and engaging approaches to youth mental health assessment, potentially lowering textual and cognitive barriers while retaining emotional nuance.

### Participatory innovation and methodological reflections

This project extends participatory methodologies into the domain of psychometric tool design, a space traditionally governed by positivist and psychometric paradigms. Co-developing a measure with young people required negotiating between rigour and responsiveness, highlighting the methodological tension between standardisation and personalisation [8, 17, 32]. Our findings echo broader debates about the need for hybrid epistemologies in youth mental-health research, where co-development with young people is seen as integral to validity and relevance [33]. Recent evidence suggests that when young people contribute directly to the design of assessment tools, those measures become more engaging, relevant, and sensitive to diverse needs and identities. This aligns with a broader shift toward digital and multimodal approaches that prioritise accessibility and user experience and respond to concerns about cultural validity and measurement bias in cross cultural context [50]. Previous reviews have argued for youth involvement and inclusive design in mental-health research to enhance relevance and acceptability [30, 33], while evidence from animation-based studies suggests that visual storytelling can increase engagement and comprehension in digital mental-health contexts [5]. Our findings extend this argument by showing that a co-developed, animation-based measure may further promote engagement among young people with diverse needs. *Animi’s* user-preference features, like dyslexia-friendly fonts, colour contrast options, and optional audio or subtitles, reflect this inclusive design logic and offer practical ways to accommodate diverse accessibility needs, directly responding to evidence that many existing youth measures exceed recommended readability levels and hinder comprehension [26, 39].

The shift from static emoji or image-based scales to animated, co-developed visuals resonates with participatory and user-centred design principles in youth mental health research. Engaging young people as co-developers rather than passive participants enhances the personal relevance, cultural sensitivity, and emotional authenticity of resulting tools [28]. This supports ongoing or participatory methodologies that move beyond tokenistic inclusion and recognise young people as equal partners in the design and evaluation of mental-health interventions [47].

Although co-development was central to this project, it was shaped by practical and structural constraints, including project timelines, ethics procedures, and institutional expectations of deliverables. Such tensions are well documented in participatory health research, where meaningful inclusion can be limited by organisational or methodological boundaries [14, 21]. Young people’s contributions were often transformative, yet not all ideas could be implemented due to design feasibility, time constraints or safeguarding requirements. Recognising these boundaries helps avoid idealising participation and sustains transparency and trust within co-researcher relationships. These reflections on the limits of participation also prompted a rethinking of what meaningful involvement might look like beyond the research process itself, particularly in how young people relate to and manage the data they generate.

### Data ownership, agency and relational ethics

Beyond participation in design, questions of data ownership and agency emerged as central to how young people understood their relationship with mental-health measures. Many participants expressed a desire for greater access to and control over the data they generate when completing assessments, wanting not only to contribute to research but also to use that data in personally meaningful ways. This points to a wider ethical and epistemological shift: from data being extracted *about* young people to data being co-managed *with* them. Such an approach aligns with emerging debates on participatory data ethics and youth digital citizenship [30, 33]. Developing an interactive platform to accompany *Animi* could support this by allowing young people to visualise and track their emotional data over time, share insights selectively (e.g., with clinicians, teachers, or peers), and use their data to advocate for their needs. However, this raises important safeguarding and governance considerations around privacy, consent, and the potential re-use of sensitive information.

Rather than viewing these as barriers, they invite dialogue about how digital mental-health tools might balance protection with autonomy, echoing wider debates about personal data access in systems like the NHS App [11]. Recent work on digital mental-health rights highlights that user empowerment and ethical governance must evolve together,safeguarding privacy is not enough if users remain excluded from control over their own data [2]. Our findings reflect this tension. Young people involved in *Animi’s* co-development repeatedly expressed a wish to see and use their data to support self-understanding or communicate needs to trusted adults or professionals. Embedding such functionality within future iterations of *Animi* could align with calls for participatory data ethics transforming young people from data subjects into active co-interpreters of their wellbeing information. In this sense, the ethical and legal considerations identified by Afrihyia et al. [2] provide an important framework for how tools like *Animi* might responsibly navigate the line between protection and empowerment.

While technological advances offer exciting opportunities for more dynamic and interactive data collection, such tools must not reinforce individualised notions of responsibility for mental health [19]. Enabling young people to access and manage their own data should be understood not as transferring the burden of care to individuals, but as supporting collective and relational approaches to wellbeing. Tools like *Animi* are best positioned as facilitators that bridge young people, practitioners, families, and community resources, enhancing dialogue rather than replacing human contact or professional support. In this way, the measure can complement wider trauma-informed and community-based practices that recognise health as socially embedded rather than purely individual [18]. Taken together, these findings demonstrate how participatory, ethically grounded, and visually oriented methodologies can inform both conceptual and practical shifts in youth mental-health assessment.

### Clinical and conceptual implications

Clinically, *Animi* may serve as a valuable engagement tool for young people reluctant to complete conventional measures, especially in school, community, or primary-care settings. Its interactive and accessible design could help practitioners open conversations about distress and wellbeing using a more relatable visual language. However, digital tools like *Animi* require practitioner training to interpret visual and affective data responsibly, ensuring that animation-based measures complement rather than replace clinical dialogue. As digital mental-health tools continue to expand, co-developed and visually oriented approaches like *Animi* may help bridge experiential and clinical understandings of youth mental health.

### Strengths and limitations

While a highly inclusive and rigorous methodology was employed, several limitations remained. Despite the diversity of participants and the digital tool's ability to address many barriers to using text-based or other image-based measures, some groups were still identified as excluded from the measure (e.g., blind young people, non-verbal young people, and those experiencing digital poverty). Due to the time constraints of the project, we were unable to examine the psychometric properties of the newly developed tool. Participants suggested improving the audio descriptions, providing information for professionals supporting young people to complete the measure, and creating a paper version of the measure to be more inclusive of these groups. Nonetheless, the extent to which these groups are fully included may be limited.

The meaningful co-production and impact that focus group participants and young co-researchers had on the measure were a strength of the present research. Young co-researchers found the experience rewarding and enjoyed the unique opportunity to provide their opinions reflectively, without a right or wrong answer. This research drew heavily on young people’s voices, which is crucial to the Lundy model, and enabled them to learn beneficial new skills (e.g., qualitative training and the CBAR approach). The opportunity for young co-researchers to socialise with others in a safe and inclusive environment was significant, given that these young people belonged to marginalised and minoritised groups that are often not afforded such spaces and opportunities.

## Conclusions and next steps

This work successfully created a usable and accessible digital measure to assess depression and anxiety in young people through an inclusive and interactive process drawing heavily on the voices of young people, which has not previously been done. *Animi* is unique in its use of co-designed animated characters to represent both the presence and absence of mental health symptoms. Unlike existing text or emoji-based measures, *Animi* integrates contemporary digital animation, user preference settings, and interactive response formats. *Animi* is one of the first co-produced animation-based mental health measures designed with and for young people and prioritising accessibility, representation, and interactive engagement. Subsequent work will further revise the measure and animations based on the provided feedback. A larger study examining the measures' psychometric properties, including reliability, validity, and feasibility for different ages and contexts, will then be necessary to allow for wider usage of this novel and accessible measure for depression and anxiety in young people. There is particular applicability for school settings and clinical settings, and it can help open conversations with young people about their mental health and provide a valuable metric for assessing change in anxiety and depression over time in a more accessible, acceptable, and engaging way. We hope this research can be used by others seeking to increase the accessibility, inclusivity, and representativeness of mental health measures for young people and provide valuable insight into participatory methodologies that give voice to young people.

## Data Availability

The qualitative datasets supporting the conclusions of this article are not publicly available to prevent participant re-identification, in line with our ethical approval. For further information about accessing the raw data, please contact Julian.Childs@ucl.ac.uk.
